# Silencing FOXP2 reverses vemurafenib resistance in BRAF^*V600E*^ mutant papillary thyroid cancer and melanoma cells

**DOI:** 10.1007/s12020-022-03180-y

**Published:** 2022-11-04

**Authors:** Suyuan Jiang, Yuxin Huang, Yuan Li, Qin Gu, Cuiping Jiang, Xiaoming Tao, Jiao Sun

**Affiliations:** Department of Endocrinology, Huadong Hospital Affiliated to Fudan University, Shanghai, 200040 People’s Republic of China

**Keywords:** Papillary thyroid cancer, Melanoma, BRAF mutant, VEM resistance, FOXP2

## Abstract

**Background:**

Vemurafenib (VEM) is a commonly used inhibitor of papillary thyroid cancer (PTC) and melanoma with the BRAF^*V600E*^ mutation; however, acquired resistance is unavoidable. The present study aimed to identify a potential target to reverse resistance.

**Materials and methods:**

A VEM-resistant PTC cell line (B-CPAP/VR) was established by gradually increasing the drug concentration, and a VEM-resistant BRAF^*V600E*^ melanoma cell line (A375/VR) was also established. RNA sequencing and bioinformatics analyses were conducted to identify dysregulated genes and construct a transcription factor (TF) network. The role of a potential TF, forkhead box P2 (FOXP2), verified by qRT-PCR, was selected for further confirmation.

**Results:**

The two resistant cell lines were tolerant of VEM and displayed higher migration and colony formation abilities (*p* < 0.05). RNA sequencing identified 9177 dysregulated genes in the resistant cell lines, and a TF network consisting of 13 TFs and 44 target genes was constructed. Alterations in FOXP2 expression were determined to be consistent between the two VEM-resistant cell lines. Finally, silencing FOXP2 resulted in an increase in drug sensitivity and significant suppression of the migration and colony formation abilities of the two resistant cell lines (*p* < 0.05).

**Conclusions:**

The present study successfully established two VEM-resistant cell lines and identified a potential target for VEM-resistant PTC or melanoma.

## Introduction

Thyroid carcinoma (TC) is the most common malignancy of the endocrine system. As of 2020, the incidence of TC has continually increased, and approximately 4.1% of patients with TC are expected to die from malignancy [1]. Papillary thyroid carcinoma (PTC) is the major histological type of differentiated thyroid carcinoma, accounting for 75–85% of all TC cases [2]. Although the estimated five year survival rate of PTC is approximately 98%, more than 25% of patients with PTC are at risk of recurrence post-surgery during long-term follow-up [3]. The point mutation of a valine-to-glutamate at residue 600 (V600E) of BRAF is the most frequent genetic variation in PTCs, accounting for 37–50% [4]. The BRAF^*V600E*^ mutant functions as the major driver of the MAPK pathway and is involved in the secondary genetic alteration of members of the PI3K-AKT pathway, thus leading to the aggressive development of PTC [5, 6]. Many studies have indicated that the BRAF^*V600E*^ mutation is associated with an increased risk of lymph node metastasis and recurrence [7, 8]. Notably, the positive rate of BRAF mutations in recurrent or metastatic PTCs is nearly 80% [9]. Targeting the BRAF^*V600E*^ mutant has thus become an important strategy in the treatment of advanced recurrent, or metastatic PTCs.

Vemurafenib (VEM) is the first orally available selective inhibitor of BRAF^*V600E*^ approved by the FDA (Food and Drug Administration) has no antiangiogenic properties for the treatment of *BRAF*^*V600E*^-TC and melanoma [10–12]. Many clinical VEM treatments for patients with the BRAF^*V600E*^ mutation have been conducted, and it was found that VEM helped some patients achieve better outcomes, especially in metastatic or unresectable PTCs refractory to radioactive iodine [13]. However, VEM resistance was found in many BRAF^*V600E*^ mutant patients within 3–12 months of treatment [14]. Data obtained clinical research and in vitro studies support the conclusion that primary or secondary resistance to VEM may result from the inhibition of apoptosis via inhibition of the B-cell CLL/lymphoma 2 (BCL2) pathway [15]. Other studies have revealed that the loss of key effectors of different pathways, including the BCL2 and PI3K/AKT/mTOR pathways, is linked to VEM resistance, and combining the BCL2 inhibitor obatoclax with VEM improved sensitivity [16]. Furthermore, simultaneous mutations in BRAF^*V600E*^ and PI3KCA are significantly associated with VEM resistance [17].

Forkhead box P2 (FOXP2) is a member of the FOXP transcription factor (TF) family and contains a C-terminal Winged-helix/Forkhead DNA binding domain, thus playing important roles in embryonic development and cancer progression [18]. FOXP2 is expressed in various cancers and acts as an oncogene or suppressor in carcinogenesis. For instance, FOXP2 can inhibit epithelial-mesenchymal transition by activating the transcription of E-cadherin and PHF2 in breast cancer cells [19]. Conversely, it is an oncogene in triple-negative breast cancer [20]. A previous study indicated that FOXP2 is decreased in TC tissues, and overexpression of FOXP2 hampers the proliferation and stemness of TC cells [21]. However, whether FOXP2 is involved in the acquired resistance to VEM remains unknown.

In this study, we aimed to identify a potential target for reversing VEM resistance based on the establishment of VEM-resistant cell lines. Because only one PTC cell line (B-CPAP) with the BRAF^*V600E*^ mutant could be established, a melanoma cell line (A375) carrying the BRAF^*V600E*^ mutant was also considered or the development of potential targets. The two VEM-resistant cell lines (B-CPAP/VR and A375/VR) were established by gradually increasing the drug concentration, followed by phenotypic detection. Next, RNA sequencing and bioinformatic analysis were performed to identify the specific effectors that reversed VEM resistance. Finally, FOXP2 was screened, and the role of FOXP2 in reversing VEM resistance was investigated.

## Materials and methods

### Cell culture and main reagents

The PTC cell line carrying the BRAF^*V600E*^ mutant, B-CPAP (Cell Bank of the Chinese Academy of Sciences, Shanghai, China), was cultured in RPMI-1640 (Gibco, Grand Island, NY, USA) supplemented with 1% penicillin-streptomycin (Gibco) and 1% NAEE (Gibco). A375 cells (Fuheng Biotech. Ltd. Co., Shanghai, China) is a melanoma cell line with the BRAF^*V600E*^ mutation and was cultured in DMEM supplemented with 2 mM glutamine (Gibco). The media for the two cell lines were supplemented with 10% fetal bovine serum (FBS; Gibco) and 1% penicillin/streptomycin (Gibco), and the cells were maintained in an incubator with 5% CO_2_ at 37 °C.

### The establishment of VEM-resistant cell lines

The VEM-resistant B-CPAP cell line (B-CPAP/VR) and the VEM-resistant A375 cell line (A375/VR) were established by gradually increasing the concentration of VEM. The induction concentration was determined from the half-maximal inhibitory concentration (IC_50_) values of the cell lines: 7 μM and 1 μM for B-CPAP and A375, respectively. After the cells resumed normal growth speed and reached 80% confluence with the addition of VEM, the next round of treatment began. The concentration of VEM was gradually increased during each round of induction. After four months of treatment, B-CPAP and A375 could survive and proliferate in a cell culture system containing 30 μM and 15 μM of VEM, respectively, and were named B-CPAP/VR and A375/VR, respectively. In contrast to the parental cells, the culture system of the two resistant cell lines required 5 μM VEM.

### Cell viability assay and half-maximal inhibitory concentration calculation

B-CPAP, A375, B-CPAP/VR, and A375/VR cell lines were collected and seeded at 5 × 10^3^ cells/well in 96-well plates and then treated with different concentrations of VEM (0, 0.01, 0.1, 1, 10, 50, and 100 μM). Four replicate wells were used for each concentration. After drug treatment for 72 h, cells were incubated with 3-[4,5-dimethylthiaoly]-2,5-diphenyltetrazolium bromide (MTT; Sigma-Aldrich) for another 3 h. Next, the supernatant was removed and dimethyl sulfoxide (DMSO; Sigma-Aldrich) was added to each well and incubated for 15 min. The optical density (OD) of each well was measured at 490 nm using a microplate reader (Thermo Fisher Scientific, Waltham, MA, USA). The survival rate (%) was calculated according to the following formula [22]:

Survival rate (%) = mean OD of experimental group/mean OD of control group × 100

The IC_50_ was calculated according to the survival rate using SPSS version 17.0. The drug resistance index was calculated according to the formula [22]:

Resistance index (RI) = IC_50_ of drug-resistant cell line/ IC_50_ of corresponding parental cell line

### VEM resistant cell lines proliferation potential assay

The B-CPAP, A375, B-CPAP/VR, and A375/VR cell lines were collected and seeded at 3 × 10^3^ cells/well in 96-well plates. The OD of each well was measured at 490 nm using the MTT assay every day for 5 days. Four replicate wells were used for each time point. The proliferation rate was calculated using the following formula [23]:

Proliferation rate = mean OD of day measured (n)/mean OD of first day (*n* = 1–5)

### Transwell assay

A Boyden chamber (8 μm; CytoSelect) inserted into a 24-well plate was used to measure cell motility. The B-CPAP, A375, B-CPAP/VR, and A375/VR cell lines were collected and resuspended in FBS-free culture medium, seeded in a chamber at 2 × 10^4^ cells/well and cultured for 24 h at 37 °C. The cells on the lower surface were fixed with 4% paraformaldehyde at room temperature for 30 min and stained with crystal violet for 20 min, followed by washing with PBS buffer and air drying. Cells passing through the lower chamber were observed under a microscope and the number of cells was recorded in six random fields. Experiments were performed in triplicate.

### Clone formation assay

B-CPAP, A375, B-CPAP/VR, and A375/VR single cells resuspended in the required medium were counted, and 800 cells/well were seeded in 6 well plates. Three days later, VEM (2 μM) was added to the medium and cells were cultured for an additional 11 days. After cloning, the cells were fixed with 4% paraformaldehyde for 30 min and stained with crystal violet for 10 min. Finally, cells were washed twice with PBS for two times and photographed using a digital camera.

### RNA sequencing

Total RNA of B-CPAP, A375, B-CPAP/VR, and A375/VR was extracted using TRIzol reagent (Invitrogen, Carlsbad, CA, USA) according to the manufacturer’s instructions. RNA quality and concentration were determined from OD260/280 readings using a NanoDrop ND-1000 spectrophotometer (NanoDrop Technologies, Montchanin, DE, USA) and assessed on a 1% gel electrophoresis. RNA samples were subjected to BHBIO (Shanghai Biotechnology Corporation, Shanghai, China) for RNA sequencing. Differentially expressed genes (DEGs) were screened under a threshold of log_2_ fold change (absolute) |Log_2_ FC | > 1 (as *n* = 2, *p*-value was not included), and the resulting genes were used for further bioinformatics analyses.

### Bioinformatic analysis

Kyoto Encyclopedia of Genes and Genomes (KEGG) analysis was conducted to analyze the enriched signaling pathways of the DEGs. A gene number > 2 was used as a threshold when screening for relevant KEGG pathways. TF-binding networks were constructed using the top 2000 dysfunctional genes utilizing weighted correlation network analysis (WGCNA).

### Cell transfection

B-CPAP/VR and A375/VR cells (1 × 10^5^ cells/well) were seeded into six-well plates. After the cells grew to 80% confluence, si-FOXP2 and control siRNA (si-NC) were transfected into VEM-resistant cell lines using Lipofectamine® 3000 (Invitrogen), according to the standard protocol. Forty-eight hours after transfection, transfection efficiency was determined by quantitative real-time PCR (qRT-PCR). The siRNA sequences for si-FOXP2 were as follows: sense (5′-3′): GGCUAGACCUCACUACUAATT, anti-sense (5′-3′): UUAGUAGUGAGGUCUAGCCTT.

### Quantitative real-time PCR (qRT-PCR)

Total RNA was extracted from the cells using a Universal RNA Extraction kit (TaKaRa, Dalian, China) and then reverse-transcribed using a PrimeScript RT Reagent Kit with gDNA Eraser (TaKaRa) according to the manufacturer’s instructions. The complementary DNA template was amplified by qRT-PCR using SYBR Premix Ex Taq (TaKaRa Bio). qRT-PCR was conducted using the StepOne Plus Real-Time PCR System (Thermo Fisher Scientific). Briefly, the reaction system for quantification included 0.5 µL cDNA, 1 µL primers, 5 µL SYBR Green, and 3.5 µL deionized water. Amplification was performed as follows:

94 °C for 5 min and 40 cycles of 94 °C for 5 s and 60 °C for 1 min. Gene expression data were analyzed using the 2^−∆∆Ct^ method and were normalized to GAPDH expression. The primer sequences synthesized by Sangon Biotech (Shanghai, China) are listed in Table 1.

**Table 1:** Primer sequences used for qPCR

genes		primer sequence
NR4A1	Forward	ATGCCCTGTATCCAAGCCC
	Reverse	GTGTAGCCGTCCATGAAGGT
SOX10	Forward	CCTCACAGATCGCCTACACC
	Reverse	CATATAGGAGAAGGCCGAGTAGA
NR4A2	Forward	GTTCAGGCGCAGTATGGGTC
	Reverse	CTCCCGAAGAGTGGTAACTGT
FOXO6	Forward	ACCTCATCACCAAAGCCATC
	Reverse	GTGCAGCGACAGGTTGTG
NFATC2	Forward	GAGCCGAATGCACATAAGGTC
	Reverse	CCAGAGAGACTAGCAAGGGG
FOXD2	Forward	CTACTCGTACATCGCGCTCA
	Reverse	TCTTGACGAAGCAGTCGTTG
FOXP2	Forward	AGGCTTCCAGTCTGTGCTGT
	Reverse	TTTGCAGCTGTAGCCTTTGA
GATA3	Forward	GCCCCTCATTAAGCCCAAG
	Reverse	TTGTGGTGGTCTGACAGTTCG
GAPDH	Forward	GGAGCGAGATCCCTCCAAAAT
	Reverse	GGCTGTTGTCATACTTCTCATGG

### Statistical analysis

SPSS statistical software was used for statistical analysis. The data were tested using the Students’ t-test to analyze the differences between the different groups. Statistical significance was set at *p* < 0.05.

## Results

### Establishment of VEM-resistant B-CPAP and A375 cell lines

**Fig. 1:**
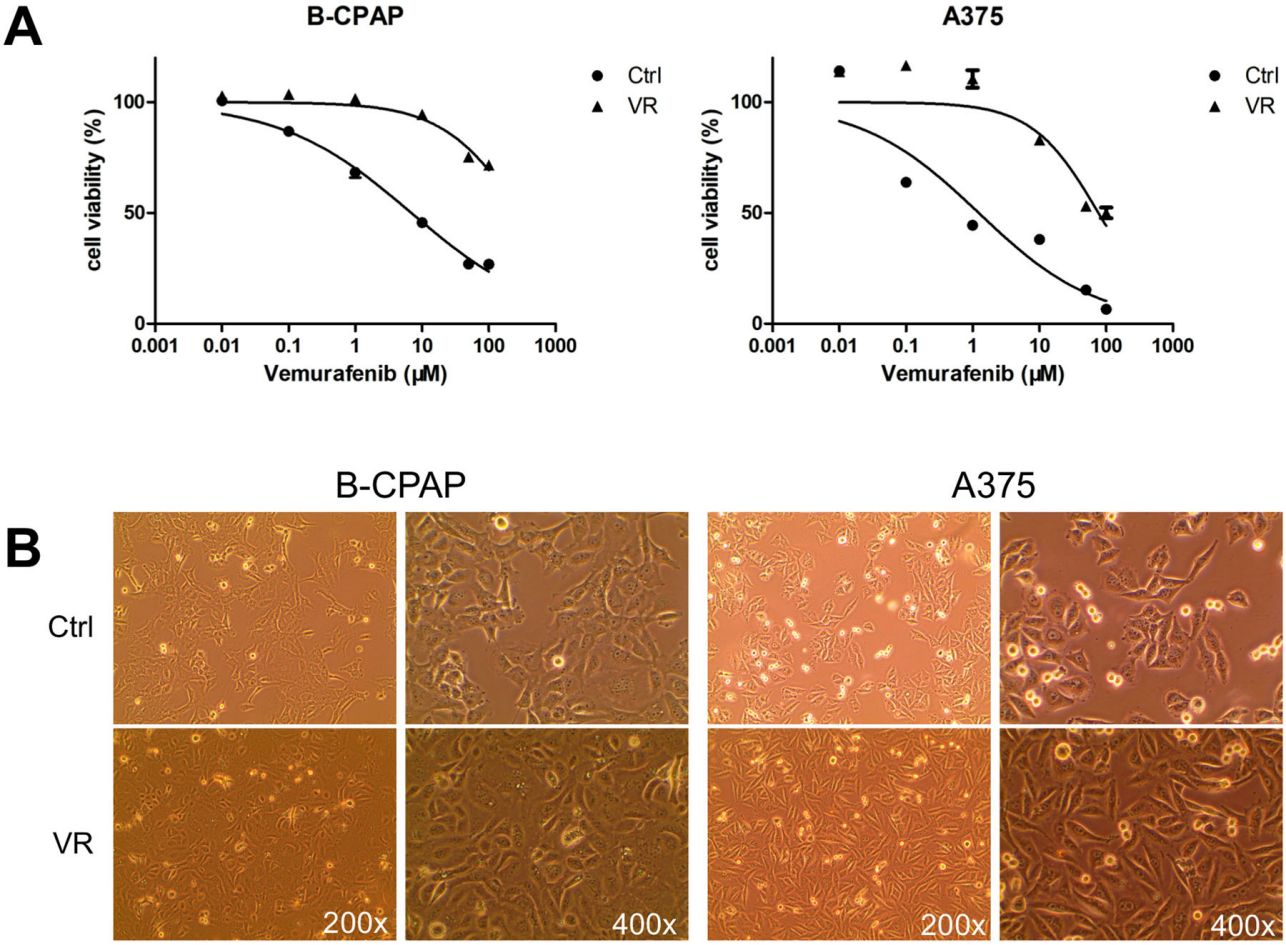
The establishment of VEM-resistant cell lines. **A** The morphological differences between VEM-resistant and parental cell lines. **B** The drug sensitivity of VEM-resistant and parental cell lines was detected by MTT assay

To establish VEM-resistant cell lines, the concentration of VEM treatment was gradually increased from 7 to 30 μM for B-CPAP and 1 to 15 μM for A375. After four months, the IC_50_ value of B-CPAP/VR increased to 304.3 μM, around 44 times of the value of parental B-CPAP (6.92 μM). IC_50_ values of A375/VR and A375 were 76.76 μM and 1.23 μM respectively, and the resistance index was 62.3 (Fig. 1A). Morphological changes were observed between the VEM-resistant and parental cell lines. The size of the B-CPAP/VR cells increased slightly, and a sickle-like shape was induced in some of the cells. Most of the A375/VR cells exhibited a spindle-shaped and elongated appearance, which was remarkably different from that of the parental A375 cells (Fig. 1B). These morphological features of the established VEM-resistant cell lines indicate a potential enhancement in cell migration ability.

**Fig. 2:**
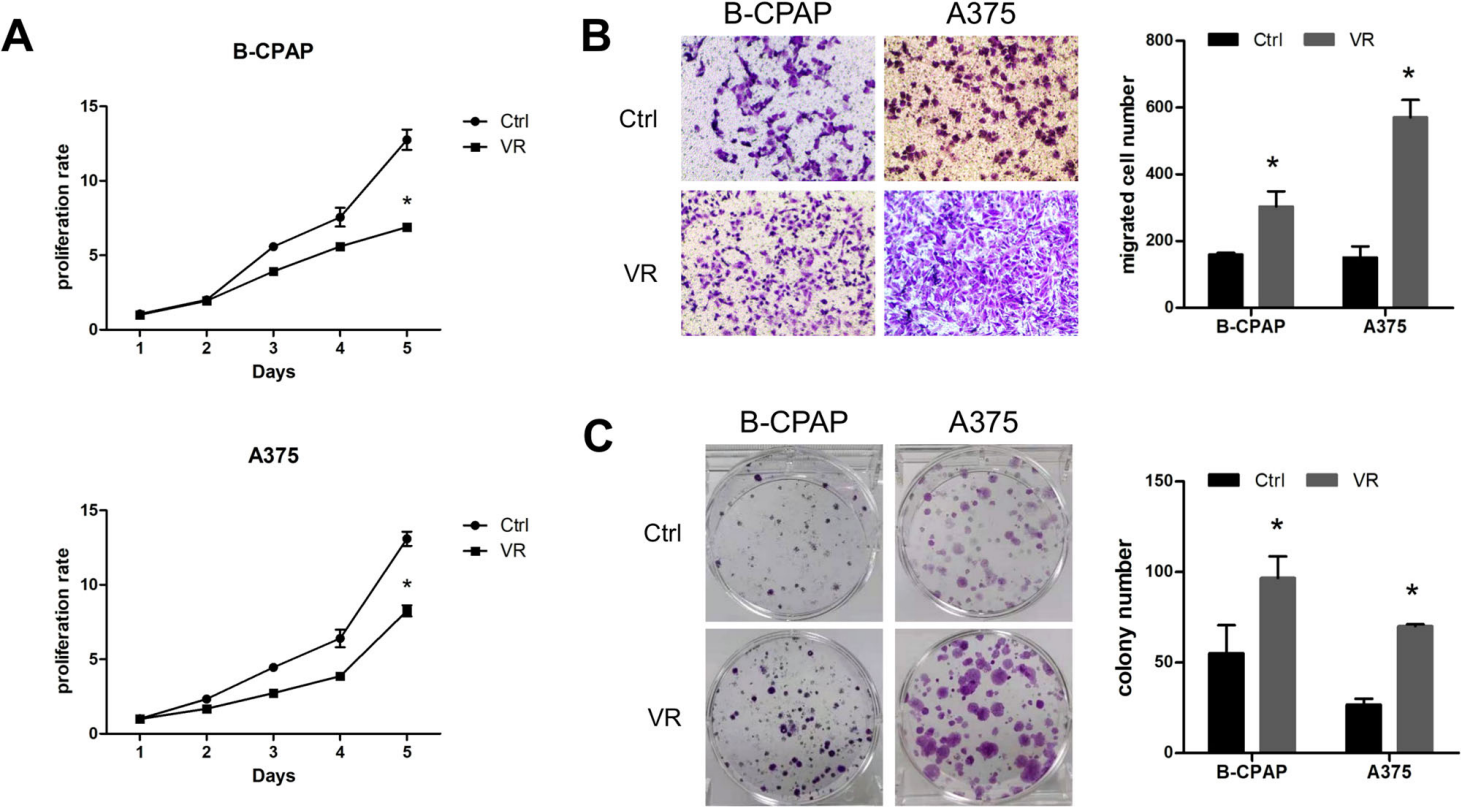
Phenotype detection of VEM -resistant cell lines. **A** The proliferation ability of different cell lines was detected using the MTT assay. **B** Transwell assay was used to test the migration ability of the cell lines. **C** Colony formation assay was used to detect the colony formation ability of the four different cell lines

The MTT assay results indicated that the proliferation ability of B-CPAP/VR and A375/VR was reduced when compared to the original cell lines (Fig. 2A, *p* < 0.05). A transwell assay was performed to evaluate cell motility. A higher number of B-CPAP/VR and A375/VR cells traversed the membrane than B-CPAP and A375 cells, respectively, indicating that the migration ability of resistant cells was significantly enhanced (Fig. 2B, *p* < 0.05). The colony formation assay results indicated that the colony number of both B-CPAP/VR and A375/VR was more than twice that of their parental cell lines after treatment with VEM for 11 d (Fig. 2C, *p* < 0.05). All phenotypic changes suggested that the VEM-resistant cell lines were successfully established.

### DEG profiles of VEM sensitive and resistant cells

**Fig. 3:**
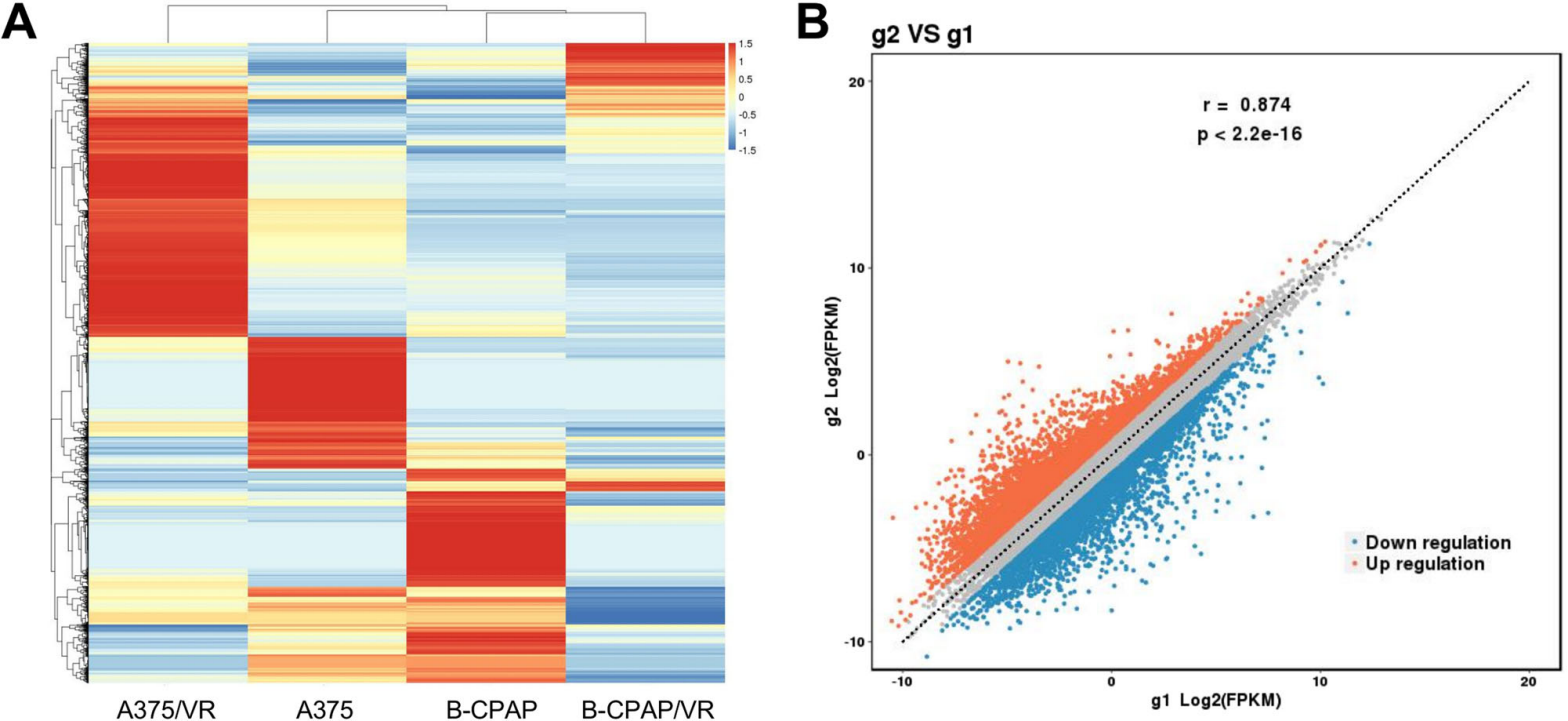
RNA sequencing analysis of DEGs in VEM-resistant cell lines. **A** Heat map of DEGs. Each column represents a sample, and each row represents a gene. Red indicates up-regulated, and blue indicates down-regulated. **B** Scatter plot of DEGs. g2: VR group; g1: Ctrl group. Screening of DEGs: |Log_2_ FC | å 1. (*n* = 2, therefore the *p*-value is not considered). DEGs, differentially expressed genes; FC Fold change

To explore the gene changes in VEM-sensitive and-resistant cells, the key genes involved in drug-resistant functional pathways were identified using RNA sequencing technology. In total, 9177 DEGs, including 5273 upregulated and 3904 downregulated genes, were screened from the resistant group with a | Log_2_ FC | > 1 threshold. As the number of samples in each group was two, the p-values were not taken into consideration. The clustered heat map indicated that the up-/downregulation distribution of DEGs was not consistent between B-CPAP/VR and A375/VR (Fig. 3A). Scatter plots also show the DEGs screened from the resistant group (Fig. 3B). The top 10 up- and downregulated DEGs are summarized in Table 2. It should be noted that the FC of the 10 DEGs were all more than 300, e.g., KRT6B was downregulated by > 1500-fold and RARRES2 was upregulated by > 900-fold (Table 2).

**Table 2:** The top 10 upregulated or downregulated DEGs in resistant group listed by fold change

	gene id	gene name	log2FC	log2FC abs	FC abs	Up/down
1	ENSG00000185479	KRT6B	−10.605	10.605	1557.323	DOWN
2	ENSG00000148346	LCN2	−10.104	10.104	1100.597	DOWN
3	ENSG00000106538	RARRES2	9.944	9.944	984.943	UP
4	ENSG00000143171	RXRG	−9.577	9.577	763.969	DOWN
5	ENSG00000168542	COL3A1	9.271	9.271	617.696	UP
6	ENSG00000116996	ZP4	−8.870	8.870	467.895	DOWN
7	ENSG00000107295	SH3GL2	8.623	8.623	394.294	UP
8	ENSG00000088992	TESC	−8.481	8.481	357.231	DOWN
9	ENSG00000107242	PIP5K1B	8.399	8.399	337.671	UP
10	ENSG00000151952	TMEM132D	−8.334	8.334	322.796	DOWN

### KEGG pathway and classification analyses

**Fig. 4:**
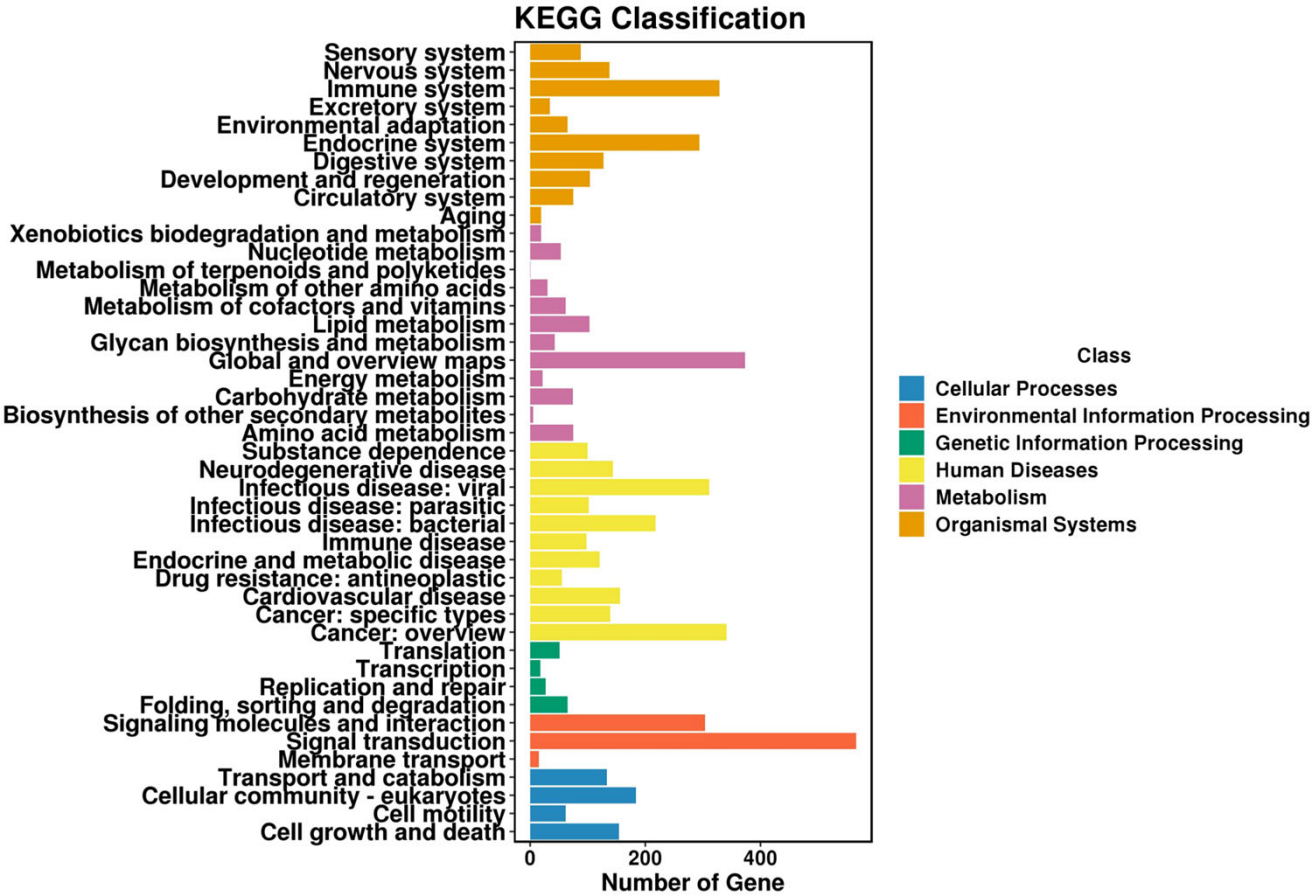
Kyoto Encyclopedia of Genes and Genomes (KEGG) pathway analysis. KEGG pathway analysis was conducted to determine the involvement of DEGs in different biological pathways. The bar indicates the number of DEGs. The color of the bar indicates the classification of KEGG pathway. DEGs Differentially expressed genes

KEGG pathway and classification analyses were performed for the DEGs to evaluate their roles in different biological pathways. The result indicated that, 341 pathways were involved, which may be related to the reduced cell proliferation rate and increased migration ability (Fig. 4). Signal transduction was one of the major pathways, of which 566 DEGs were included. Other identified genes were classified as cell growth and death (154) and cell motility (62). The distribution of pathways is shown in Table S1.

### TF regulatory network construction

**Fig. 5:**
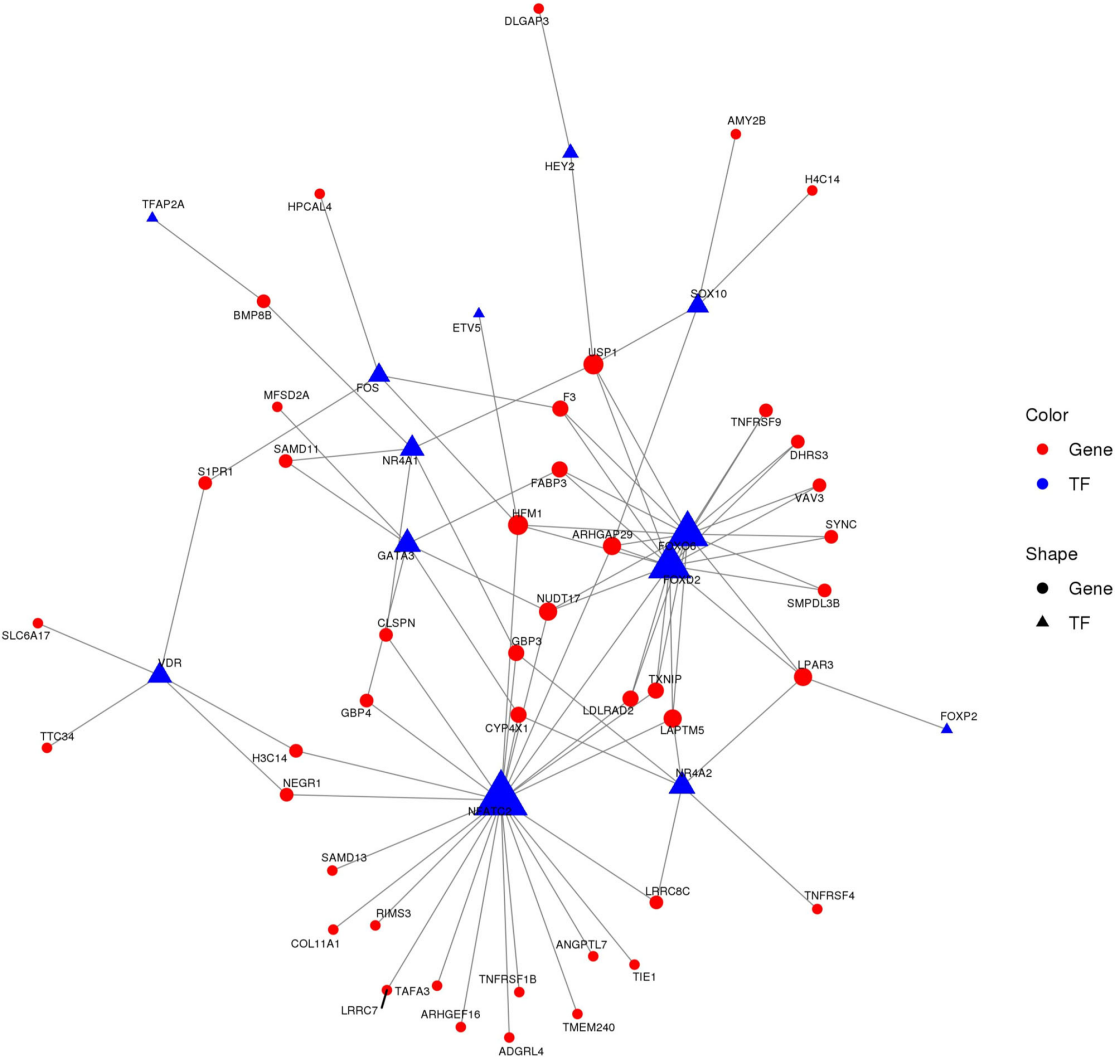
Diagram of transcription factor regulatory network. Top 2000 DEGs were used to construct a transcription factor regulatory network diagram

We constructed a TF regulatory network to uncover connections among the DEGs. Since all DEGs contained many TFs and target genes, the top 2000 DEGs were selected for the construction of the TF regulatory network. We found 13 TFs and 44 targeted genes, including NFATC2 with 25 targeted genes and FOXD2 with 16 targeted genes (Fig. 5). The fold-changes of the 13 TFs are listed in Table 3.

**Table 3:** Expression changes of 13 dysregulated TFs in TF network diagram

	gene id	gene name	log2FC	log2FCabs	FCabs	up/down
1	ENSG00000123358	NR4A1	−5.269	5.269	38.548	DOWN
2	ENSG00000100146	SOX10	−3.703	3.703	13.026	DOWN
3	ENSG00000153234	NR4A2	−3.103	3.103	8.593	DOWN
4	ENSG00000204060	FOXO6	2.802	2.802	6.975	UP
5	ENSG00000101096	NFATC2	−2.684	2.684	6.428	DOWN
6	ENSG00000135547	HEY2	−2.479	2.479	5.573	DOWN
7	ENSG00000111424	VDR	−2.349	2.349	5.094	DOWN
8	ENSG00000170345	FOS	−2.046	2.046	4.130	DOWN
9	ENSG00000186564	FOXD2	−2.046	2.046	4.129	DOWN
10	ENSG00000244405	ETV5	−1.963	1.963	3.897	DOWN
11	ENSG00000137203	TFAP2A	1.898	1.898	3.726	UP
12	ENSG00000128573	FOXP2	1.886	1.886	3.697	UP
13	ENSG00000107485	GATA3	1.871	1.871	3.659	UP

### Validation of the dysregulated TFs by qRT-PCR

**Fig. 6:**
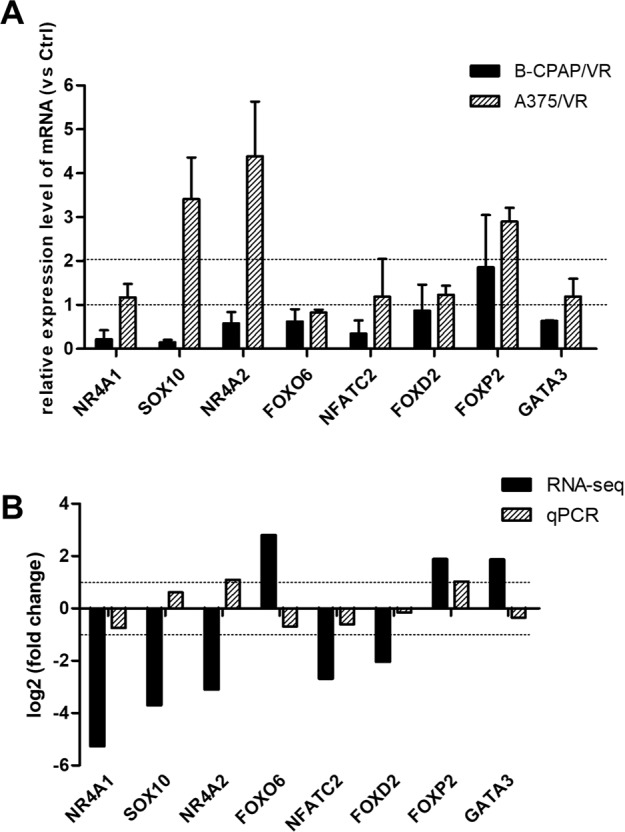
Validation of the dysregulated TFs by qRT-PCR. **A** qRT-PCR was conducted to detect the relative expression levels of TFs in B-CPAP/VR and A375/VR (vs corresponding control cells). **B** The Log_2_ FC of TFs detected by qRT-PCR in the VEM-resistant group (vs RNA sequencing). The dotted line indicates the position of |Log_2_ FC | = 1. FC: fold change. TF Transcription factors

Eight TFs, including the top five TFs and three TFs randomly chosen from the remaining eight TFs, were selected for qRT-PCR validation among the 13 TFs. qRT-PCR results indicated that only the relative expression level of FOXP2 was consistent in the two resistant cell lines, whereas the other seven showed an opposite expression change (Fig. 6A). In addition, qRT-PCR data for NR4A1, NFATC2, and FOXP2 were consistent with the RNA sequencing results (Fig. 6B). Combining the results shown in Fig. 6A, B, FOXP2 was selected for further investigation.

### Silencing FOXP2 increased the sensitivity of VEM resistant cell lines

**Fig. 7:**
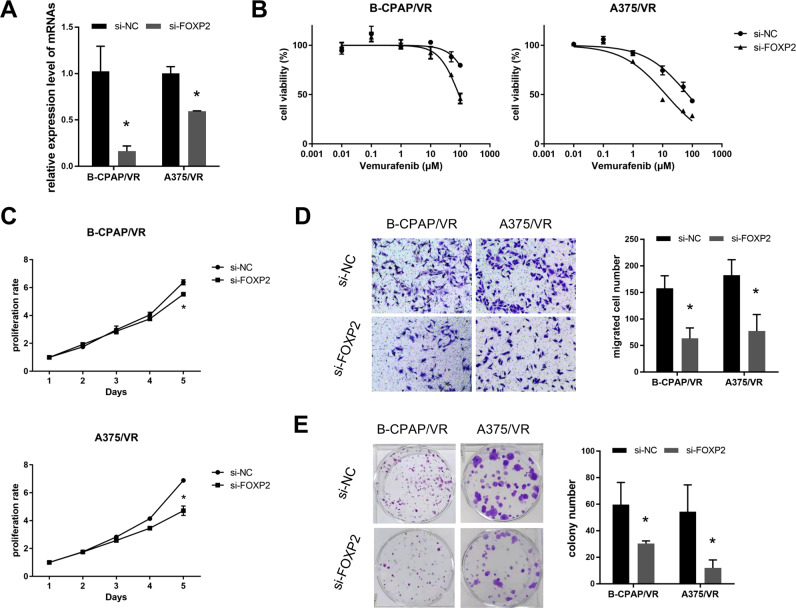
Silencing FOXP2 increases the sensitivity of resistant cells to VEM. **A** qRT-PCR was performed to validate the transfection effects. **B** Sensitivity of drug-resistant cell lines to VEM was detected using the MTT assay. **C** Proliferation ability was detected by MTT assay. **D** Transwell assays was performed to detect cell migration ability. **E** Colony formation assay was performed to evaluate the cell clone formation ability

The expression level of FOXP2 in B-CPAP/VR and A375/VR were knocked down using siRNA. qRT-PCR results indicated a significant decrease in FOXP2 expression in the si-FOXP2 group compared to that in the si-NC group (Fig. 7A, *p* < 0.05). MTT analyses showed that after knockdown FOXP2, the IC_50_ values of B-CPAP/VR and A375/VR were reduced to 90.77 and 13.05 µM, respectively, much lower than the si-NC group cells (291.3 and 70.9 µM, respectively) (Fig. 7B). In addition, the proliferation rate of cells in the si-FOXP2 group was reduced compared to that in the si-NC group (Fig. 7C, *p* < 0.05), and the FOXP2 knockdown group showed weaker clone formation and migration abilities (Fig. 7D, E, *p* < 0.05). All the phenotype assays indicated that after downregulating FOXP2 expression, the VEM-resistant cell lines B-CPAP/VR and A375/VR were more sensitive to VEM.

### Prediction of downstream pathways regulated by FOXP2

To reveal the possible mechanism and role played by FOXP2 in VEM resistance, we searched for FOXP2 in Table S1, but no relevant pathway was found. Next, we focused on the target gene, lysophosphatidic acid receptor 3 (LPAR3), of FOXP2, which might establish some connections with the pathways. LPAR3 was involved in five pathways (Table 4); interestingly, three of these pathways were involved in the signal transduction. These three signaling pathways may be the key downstream pathways for FOXP2 to reduce VEM resistance, which requires further validation.

**Table 4:** Prediction of downstream pathways regulated by FOXP2, KEGG classification analysis of LPAR3

pathway_3	pathway_3_num	pathway_2	pathway_2_num	pathway_1	pathway_1_num
Pathways in cancer	156	Cancer: overview	341	Human Diseases	857
Phospholipase D signaling pathway	45	Signal transduction	566	Environmental Information Processing	720
PI3K-Akt signaling pathway	110	Signal transduction	566	Environmental Information Processing	720
Rap1 signaling pathway	70	Signal transduction	566	Environmental Information Processing	720
Neuroactive ligand-receptor interaction	114	Signaling molecules and interaction	304	Environmental Information Processing	720

## Discussion

Acquired drug resistance often occurs when it comes to clinical treatment for patients with cancer, especially for those carrying a mutation in a specific gene. BRAF^*V600E*^ is the most common mutation in some types of cancer. It is highly related to high mortality in patients with PTC or melanoma, which also stimulates research on BRAF inhibitors. To date, the development of new targets is still an important strategy to reverse resistance to VEM and prolong the effective treatment of drug-resistant patients.

In clinical and pre-clinical studies on BRAF inhibitor resistance, the time for VEM resistance development ranged from 3 weeks to 1 year, and very few patients survived without developing resistance [24]. It was found that RI was significantly higher in patients resistant to VEM within 3 weeks than in those resistant to VEM within 1 year. This suggests that the established resistant cell lines with higher RI in vitro may retain more typical phenotypes of the cells in the tumor, compared to the resistant cell lines with lower RI. In this study, the RI of the established VEM-resistant cell lines B-CPAP/VR and A375/VR was more than 40, which is much higher than the RI (around 3) reported in other studies [25, 26]. Primary melanoma cells isolated from patient biopsies with the BRAF mutant were first treated with a high concentration of VEM (10 μM) for 1 h, followed by four months of 1 μM VEM treatment [16]. The present study established the B-CPAP/VR and A375/VR cell lines by gradually increasing the concentrations of VEM, which was similar to the clinical VEM treatment strategy. Moreover, phenotype assays indicated that the resistant cell lines showed a slower proliferation rate and higher migration and clonal formation abilities, which were completely different from those of the sensitive cells.

According to RNA sequencing data, many of the DEGs involved in the pathways were related to human diseases, but most of them were classified in signal transduction pathways. Many reports have also revealed that signaling pathways are indeed related to drug resistance in TC and melanoma based on in vitro and clinical studies [27]. Clinical and pre-clinical research shows that activation of either or both pERK or pAKT pathways occurred in most of the patients resistant to VEM despite the variable resistance drivers [24]. The PI3K/Akt pathway is a critical molecular signaling pathway involved in carcinogenesis and acquired resistance. Interestingly, in the present study, the PI3K/Akt pathway was also linked to the acquisition of VEM-resistant cell lines B-CPAP/VR and A375/VR through the target gene of FOXP2. In addition to the PI3K/Akt pathway, the MAPK pathway and its effectors (RTK, RAS, BRAF, MEK, and ERK) also play key roles in this process. The two tumorigenesis pathways are both stimulated by the activation of RTK, which is also the target of effective inhibitors such as cabozantinib, vandetanib, sorafenib, and lenvatinib in the treatment of TC and melanoma [28]. As the existing data indicate, RTK and BRAF inhibitors are both effective for targeting BRAF^*V600E*^; however, to prevent drug resistance, more targets belonging to related pathways need to be identified.

TFs are proteins that control DNA transcripts to mRNA, which can not only upregulate downstream gene expression but also silence specific genes. Therefore, dysregulated TFs induce specific diseases, including cancer, which makes TFs interesting targets for future medications [29]. Many dysregulated pathways in VEM-resistant cells were identified as signaling transduction-related, which directed our attention to explore functional TFs. FOXP2 is one of the TFs upregulated in both the established VEM-resistant cell lines. Further phenotypic assays indicated that B-CPAP/VR and A375/VR became sensitive to VEM after silencing FOXP2. In addition, proliferation, migration, and clonal formation abilities were much weaker than those in the control group. This suggests that FOXP2 is a potential target to reverse VEM resistance. Previous studies have indicated that FOXP2 is a suppressor in TC cells [21, 30], which is different from the oncogenic role found in VEM-resistant cells. The role of FOXP2 in tumorigenesis remains controversial. For instance, FOXP2 functions as an oncogene in colorectal cancer [31] and diffuse large B-cell lymphoma [32] but also acts as a suppressor in prostate cancer [33] and gastric cancer [34]. Additionally, FOXP2 has been found to play opposite roles in breast cancer [19] and in triple negative breast cancer [20]. Therefore, it is inappropriate to define it as a complete oncogene or suppressor. The opposite roles of FOXP2 in TC and VEM-resistant cancer cells might be due to the expression changes of upstream or downstream mediators, since a large number of DEGs were identified in the VEM-resistant cells.

KEGG analysis showed that FOXP2 does not directly participate in some signaling pathways, as FOXP2 functions as a TF. Nevertheless, it is possible for FOXP2 to manipulate some downstream genes to turn on/off signal transduction, and the TF network suggests that there is a connection between LPAR3 and FOXP2. LPAR3 encodes a member of the G protein-coupled receptor family and the EDG family of proteins [35]. It is not only involved in the PI3K/Akt pathway but also in the Rap1 signaling pathway and other pathways in cancer. Therefore, LPAR3 could be a key downstream gene for FOXP2 to overcome VEM resistance, which requires further investigation.

## Conclusions

In this study, we established robust VEM-resistant BRAF^*V600E*^ mutant PTC and melanoma cell lines, which can be used as in vitro models for other VEM-resistance studies. FOXP2 is a potential target and should be further explored as an innovative medication to reverse VEM resistance. The present study also identified a large number of DEGs, thereby providing a databank for target development.

## Supplementary Information

Table S1

## Data Availability

All data generated or analyzed during this study are included in this article. Further enquiries can be directed to the corresponding author.
